# Detection of binucleated nephrin-marked podocytes by flow cytometry in the urine of patients with obesity

**DOI:** 10.1007/s40620-023-01730-9

**Published:** 2023-09-19

**Authors:** Almudena G. Carrasco, Adriana Izquierdo-Lahuerta, Ignacio González de Pablos, Rocio Vila-Bedmar, Marina Martin-Taboada, Esteban Porrini, Enrique Morales, Gema Medina-Gómez

**Affiliations:** 1 Department of Basic Health Sciences, Faculty of Health Sciences, Rey Juan Carlos University, Avenida de Atenas s/n, Alcorcon, 28922 Madrid, Spain; 2 Department of Nephrology, Hospital Universitario, 12 de Octubre, Madrid, Spain; 3 University La Laguna, Instituto Tecnologias Biomedicas (ITB), Tenerife, Spain; 4 Investigation Institute of University Hospital, 12 de Octubre, Madrid, Spain; 5 LAFEMEX, Department of Basic Health Sciences, Faculty of Health Sciences, Rey Juan Carlos University, Madrid, Spain

During glomerular vasodilation associated with obesity, podocytes detach and are shed into urine [1, 2, S1]. Although podocyte-associated mRNA has been previously found in the urine of morbidly obese patients, even at normal albuminuria levels [3], a marker for early renal damage is essential.

Analysis of podocalyxin has been used to detect podocytes [4] and vesicles [S2, S3], however this marker is not exclusive for this type of cells [5]. Moreover, podocin and nephrin mRNA have also been detected [1,S4,S5], but their clinical utility is clear[S6].

Here we present a pilot study to propose a new non-invasive method to determine the presence of podocytes in the urine of patients with obesity based on the detection of nephrin on the podocyte surface, their nuclear lamin A content, associated with the ratio between podocytes and total cells found in urine.

**Fig. 1:**
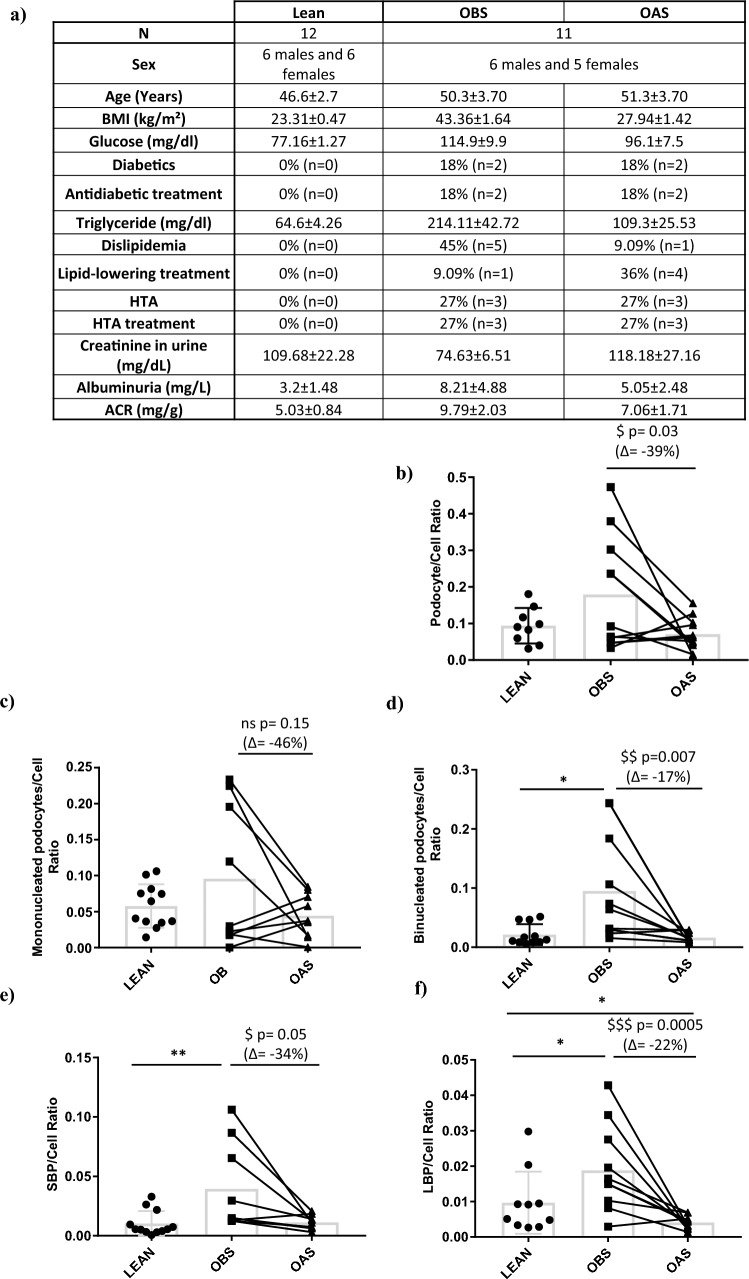
Determination of the ratio between different populations of podocytes and total cell number in 24 h urine. **a** Brief characterization of the subjects enrolled in the study and determination of the podocyte/cell ratio and percentage of change of **b** total podocyte, **c** mononucleated, **d** binucleated, **e** Small Binucleated, **f** Large Binucleated podocytes through Flow Cytometry marked by antinephrin and antilamin. *LBP* large binucleated podocytes. *SBP* small binucleated podocytes. *BMI* body mass index. *HTA* hypertension. *ACR* albumin/creatinine ratio. Grey bar indicates the mean for each group. Patients are represented individually, black square for OBS (Obese before Surgery) and black triangle for OAS (Obese after surgery). Unpaired *t* test was performed to compare OBS vs LEAN (*P ≤ 0.05; **P ≤ 0.01). Repeated measures ANOVA was used to compare OAS vs OBS (*ns* non-significant, ^$^*P* ≤ 0.01; ^$$^*P* ≤ 0.01; ^$$$^*P* ≤ 0.001)

We collected 24 h urine samples from lean subjects and patients who were morbidly obese before surgery and obese one year after bariatric surgery (Fig. 1a). We used flow cytometry, measuring nephrin to detect podocytes, and lamin A to detect binucleated podocytes (Supplementary Methods). The different podocyte populations included mononucleated podocytes, small binucleated podocytes and large binucleated podocytes (Fig. S1).

Obese before surgery patients exhibit a higher concentration of binucleated podocytes compared to lean subjects (Fig S3). Contrary to our expectations, after bariatric surgery we observed that the urinary concentration of podocytes was increased compared to what was observed before surgery (Fig. S3). Even when we normalized for volume (Fig. S4) and mg of creatinine (Fig S5) in 24 h urine, we obtained similar results.

We then questioned the reliability of volume as a normalization factor in our samples. Therefore we normalized our podocyte populations with regard to the total number of cells found in 24 h urine samples. Although there were non-significant changes in the total podocyte/total cell ratio between obese patients before surgery and lean subjects, we observed a significant reduction after surgery compared to before surgery (Fig. 1a). This reduction was primarily due to changes in binucleated podocytes, and the binucleated podocyte/total cell ratio in urine was significantly higher in obese patients before surgery compared to lean subjects (Fig. 1d). However, after surgery patients exhibited a decrease in this ratio (Fig. 1d), which aligned more with the clinical status (Fig. 1a). These changes were not observed for mononucleated podocytes (Fig. 1c).

Several attempts have been made to detect early glomerular damage through analysis of the urine [5] using non-specific podocyte proteins [4, S2, S3, 5]. Previous studies have used podocalyxin-positive cells in urinary sediment from healthy individuals and patients to quantify podocyturia. However, these studies relied on a non-specific podocyte protein, and were conducted using spot urine samples, which were then extrapolated to 24 h [6, 7]. In our study, we propose a potentially more precise method for measuring podocyturia. We measured the podocyte-specific marker nephrin to detect podocytes [3]. Unlike previous approaches, our method is based on 24 h urine samples and utilizes counting beads as a quantitative tool.

We also observed that about half of the patients presented a reduction in the pathological binucleated/cell ratio after bariatric surgery, while the other half did not (Fig. 1d). Upon analyzing both groups, we found that those who experienced a reduction in the binucleated/cell ratio after bariatric surgery were younger and had fewer or less severe comorbidities (Table S1). We hypothesized that this could be attributed to the prolonged duration of complications associated with obesity in older patients. Kidney injury could have persisted for a longer period, resulting in a greater loss of podocytes before the measurements were performed. Consequently, podocyturia could potentially serve as an indicator for detecting early stages of glomerular injury caused by obesity.

Furthermore, we observed that both small binucleated podocyte/cell and large binucleated podocyte/cell ratios were significantly higher in obese patients before surgery compared to lean subjects, while they decreased after bariatric surgery (Fig. 1e and f). We hypothesized that the number of podocytes in after surgery patients may be lower due to the absence of active kidney damage (Fig. 1f). These findings are in agreement with previously observed data [8], leading us to propose this normalization approach.

After podocyte injury, karyokinesis occurs, leading to death through mitotic catastrophe [S7]. Detecting binucleated podocytes has been a challenging task with limited success [6, 7, S8]. In this study, we propose the use of lamin A as a marker for identifying binucleated podocytes. This choice is based on the fact that non-proliferating cells may lack lamin A expression [S9], and lamin A function has been found to be affected by genetic conditions known as laminopathies, which accelerate aging [S10]. Our findings indicate that the content of lamin A is possibly associated with the DNA content, suggesting that an increase in lamin A levels may indicate podocyte karyokinesis. Importantly, this increase does not appear to be linked to apoptosis in these cells. Taken together, these data support the use of lamin A as a tool for detecting glomerular injury through the identification of binucleated podocytes.

Podocyte markers have been detected in urine, but their concentrations are often very low and can be influenced by the confounding effect of urine concentration [5]. Our study suggests a protective effect of bariatric surgery one year after surgery [8], however this effect was not maintained after normalization for the podocyte concentration. This raises the question of whether we can rely on urine volume as a normalization factor [S11]. To address this issue, we propose a new method for normalizing podocyte numbers by considering the total cells in urine. This approach would allow us to use both spot or 24 h urine, enhancing flexibility in sample collection and analysis.

Our study’s primary limitation lies in the small size of our cohort and the difficulty in recruiting morbidly obese patients without diagnosed kidney damage. Therefore, it is crucial to confirm our results in a larger cohort and investigate other pathological conditions. Nevertheless, this protocol may be considered as a suggestion for exploring podocyturia.

### Supplementary Information

Below is the link to the electronic supplementary material.

Supplementary file1 (DOCX 17 KB)

Supplementary file2 (PPTX 853 KB)
