# Supplementary material for: Detection of binucleated nephrin-marked podocytes by flow cytometry in the urine of patients with obesity
Source: J Nephrol. 2023 Sep 19;37(1):245–8. doi: 10.1007/s40620-023-01730-9 (PMC10920459; doi:10.1007/s40620-023-01730-9)
Supplement: Supplementary file 1 — Supplementary file1 (DOCX 17 KB) [file 40620_2023_1730_MOESM1_ESM.docx]

**SUPPLEMENTARY METHODS**

**Design and Sample Collection**

This study was designed to evaluate the effect of weight loss in obese patients who underwent bariatric surgery. The Clinical Research Ethics Committee of Hospital Universitario 12 de Octubre and URJC evaluated the project and considered that the necessary ethical requirements of the protocol are appropriate to carry out the study.

Lean subjects were selected according to the following criteria: (i) BMI= 20-25 kg/m2; (ii) no significant differences in age compared to obese before surgery patients. Eligibility criteria for obese before surgery patients consisted in BMI > 35 kg/m2, eGFR > 60 mL/min and/or proteinuria < 0.30 g/24 h, not receiving any medication known to interfere with the studied variables and age-matched with the lean group. Obese before surgery patients did not present renal insufficiency or proteinuria.

Lastly, 24-hour urine from 12 lean subjects (IMC= 22.82±2.38 kg/m^2^) and from 20 obese before surgery patients was collected. COVID-19 infection has detrimental effects on the follow up of these patients, so we were only able to collect 11 urine samples from patients 1 year after bariatric surgery (obese after surgery patients). This study shows the results obtained from the 11 patients that could be followed. Samples were aliquoted, centrifuged and the remaining sediment was fixed with paraformaldehyde (ThermoFisher, USA) 4%.

**Sample preparation**

Three hundred µl of fixed sample were stained with Syto13TM (ThermoFisher, USA) along with counting beads (CountBrightTM Absolute Counting Beads, Invitrogen, USA) to determine the total number of cells in the sample.

The pertinent volume of sample to obtain 200,000 cells in the test tube was separated. The sample was incubated with Triton X-100 0.2%, then with FcR blocker (RayBiotech, USA) and matching isotypes (Bioss, USA). The sample was incubated with antinephrin AlexaFluor 647 conjugated (Bioss, USA) and antilamin A FITC conjugated (Nordic-MUbio, Netherlands). Counting beads were added to the test tube and analyzed in the flow cytometer (BeckmanCoulter, USA). A size threshold to avoid vesicle detection was established through 2 µm beads (BeckmanCoulter, USA).

**Identification of populations of podocytes based on the detection of nephrin/lamin A positive populations**

We used an anti-nephrin antibody to detect podocytes by flow cytometry (Figure S1c). Within this nephrin-positive marked population, we identified three different populations of different sizes when analyzing lamin A content *vs*. size (Figure S1e). In order to identify them more clearly, we continued with the complexity for the Y axis instead of size (Figure S1f). We labeled these populations as lamin +, lamin ++ and lamin +++ (Figure S1f). After singlet screening (Figure S1g), we combined them in a single plot and analyzed the remaining events (Figure S1h). We classified them as follows - nephrin+/lamin A+ as mononucleated podocytes, and those with high lamin A content as binucleated. Since there were two populations with a higher level of lamin A, one of which was larger than the other, we referred to them as Small Binucleated Podocytes or SBPs (characterized by being marked as nephrin+/lamin A++) and Large Binucleated Podocytes or LBPs (nephrin +/lamin A+++) (Figure S1h).

To confirm the detection of binucleated podocytes, we determined that podocytes lamin A ++ and +++ exhibit a higher DNA content (Supplementary Figure S2a). In addition, and since it is mandatory for lamin A content to decrease to allow apoptosis, we carried out an experiment to relate lamin A and annexin V content and found no inverse relationship (Supplementary Figure S2b).

**Statistical analysis**

Cytometry analysis was carried out through the CXP Software (BeckamnCoulter, USA). The obtained data were analyzed using GraphPad Prism 5 software. Data are presented as mean±SEM. Statistical significance between groups was analyzed by two-way ANOVA. P < 0.05 was considered statistically significant. * p value ≤ 0.05 vs. lean subjects; ** p value ≤ 0.01 vs lean subjects; *** p value ≤ 0.001 vs lean subjects. $ p value ≤ 0.05 vs patients with obesity; $$ p value ≤ 0.01 vs patients with obesity; $$$ p value ≤ 0.001 vs patients with obesity; $$$$ p value ≤ 0.0001 vs patients with obesity.

**SUPPLEMENTARY REFERENCES**

S1. Câmara NOS, Iseki K, Kramer H, et al. Kidney disease and obesity: Epidemiology, mechanisms and treatment. Nat Rev Nephrol. 2017;13(3):181-190. doi:10.1038/nrneph.2016.191

S2. Hara M, Yanagihara T, Kihara I, et al. Apical cell membranes are shed into urine from injured podocytes: A novel phenomenon of podocyte injury. Journal of the American Society of Nephrology. 2005;16(2):408-416. doi:10.1681/ASN.2004070564

S3. Hara M, Yanagihara T, Hirayama Y, et al. Podocyte membrane vesicles in urine originate from tip vesiculation of podocyte microvilli. Hum Pathol. 2010;41(9):1265-1275. doi:10.1016/j.humpath.2010.02.004

S4. Fukuda A, Wickman LT, Venkatareddy MP, et al. Urine podocin:nephrin mRNA ratio (PNR) as a podocyte stress biomarker. Nephrology Dialysis Transplantation. 2012;27(11):4079-4087. doi:10.1093/ndt/gfs313

S5. Rodrigues PG, Bringhenti RN, Do Nascimento JF, et al. Expression patterns of podocyte-associated mRNAs in patients with proliferative or non-proliferative glomerulopathies. Int J Clin Exp Pathol. 2014;7(5):2185-2198.

S6. Sato Y, Wharram BL, Lee SK, et al. Urine Podocyte mRNAs Mark Progression of Renal Disease. Journal of the American Society of Nephrology. 2009;20(5):1041-1052. doi:10.1681/ASN.2007121328

S7. Tharaux PL, Huber TB. How Many Ways Can a Podocyte Die? Semin Nephrol. 2012;32(4):394-404. doi:10.1016/j.semnephrol.2012.06.011

S8. Mühldorfer J, Pfister E, Büttner-Herold M, Klewer M, Amann K, Daniel C. Bi-nucleation of podocytes is uniformly accompanied by foot processes widening in renal disease. Nephrology Dialysis Transplantation. 2018 May 1;33(5):796–803.

S9. Broers JLV, Ramaekers FCS. The role of the nuclear lamina in cancer and apoptosis. Adv Exp Med Biol. 2014;773:27-48. doi:10.1007/978-1-4899-8032-8_2

S10. Cenni V, Capanni C, Mattioli E, et al. Lamin A involvement in ageing processes. Ageing Res Rev. 2020;62:101073. doi:10.1016/j.arr.2020.101073

S11. Schoen T, Blum J, Paccaud F, et al. Factors associated with 24-hour urinary volume: The Swiss salt survey. BMC Nephrol. 2013;14(1). doi:10.1186/1471-2369-14-246
