# Supplementary material for: Detection of binucleated nephrin-marked podocytes by flow cytometry in the urine of patients with obesity
Source: J Nephrol. 2023 Sep 19;37(1):245–8. doi: 10.1007/s40620-023-01730-9 (PMC10920459; doi:10.1007/s40620-023-01730-9)
Supplement: Supplementary file 2 — Supplementary file2 (PPTX 853 KB) [file 40620_2023_1730_MOESM2_ESM.pptx]

## Slide 1
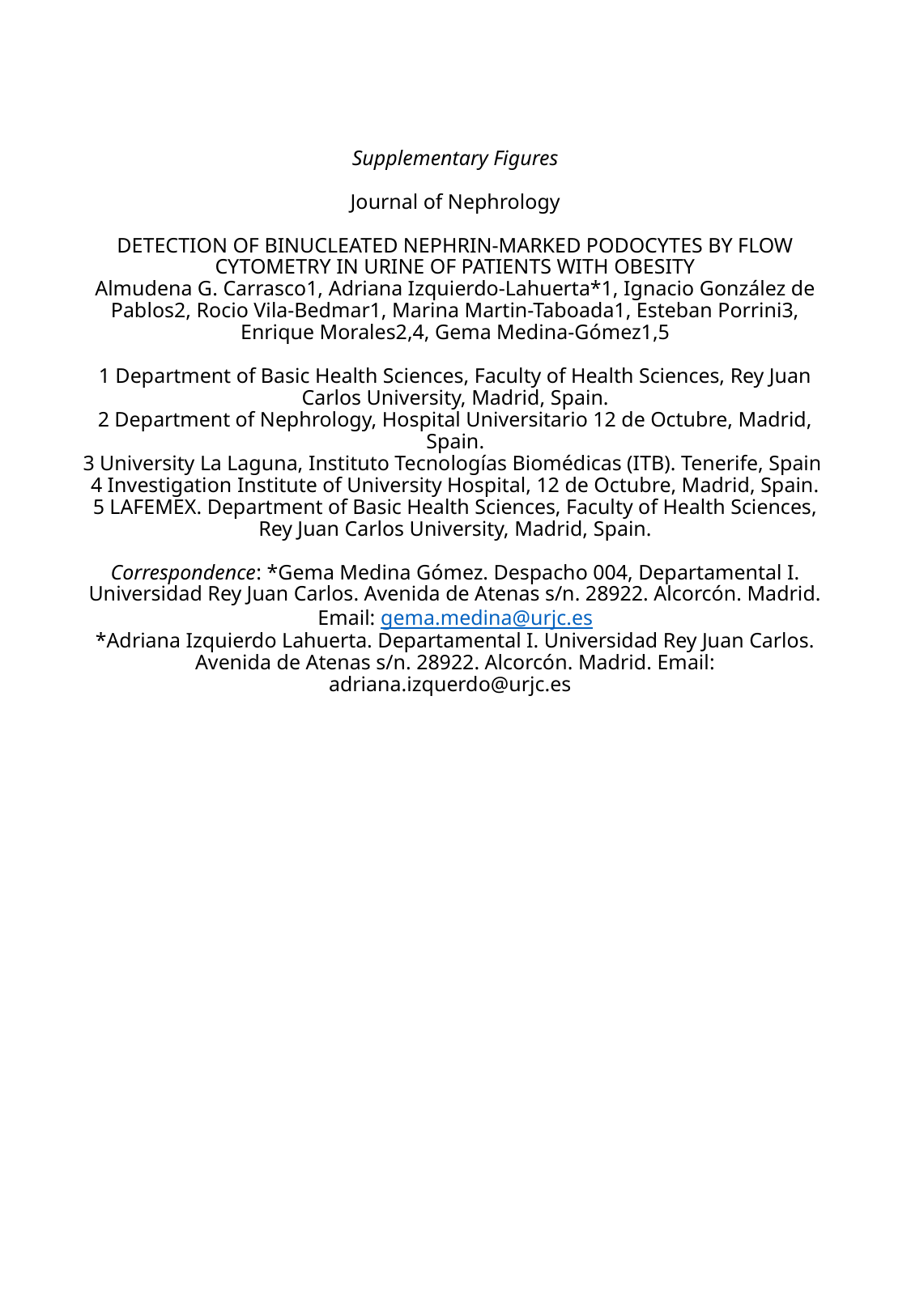

# Supplementary Figures Journal of Nephrology DETECTION OF BINUCLEATED NEPHRIN-MARKED PODOCYTES BY FLOW CYTOMETRY IN URINE OF PATIENTS WITH OBESITY Almudena G. Carrasco1, Adriana Izquierdo-Lahuerta*1, Ignacio González de Pablos2, Rocio Vila-Bedmar1, Marina Martin-Taboada1, Esteban Porrini3, Enrique Morales2,4, Gema Medina-Gómez1,5 1 Department of Basic Health Sciences, Faculty of Health Sciences, Rey Juan Carlos University, Madrid, Spain. 2 Department of Nephrology, Hospital Universitario 12 de Octubre, Madrid, Spain. 3 University La Laguna, Instituto Tecnologías Biomédicas (ITB). Tenerife, Spain 4 Investigation Institute of University Hospital, 12 de Octubre, Madrid, Spain. 5 LAFEMEX. Department of Basic Health Sciences, Faculty of Health Sciences, Rey Juan Carlos University, Madrid, Spain. Correspondence: *Gema Medina Gómez. Despacho 004, Departamental I. Universidad Rey Juan Carlos. Avenida de Atenas s/n. 28922. Alcorcón. Madrid. Email: gema.medina@urjc.es *Adriana Izquierdo Lahuerta. Departamental I. Universidad Rey Juan Carlos. Avenida de Atenas s/n. 28922. Alcorcón. Madrid. Email: adriana.izquerdo@urjc.es

## Slide 2
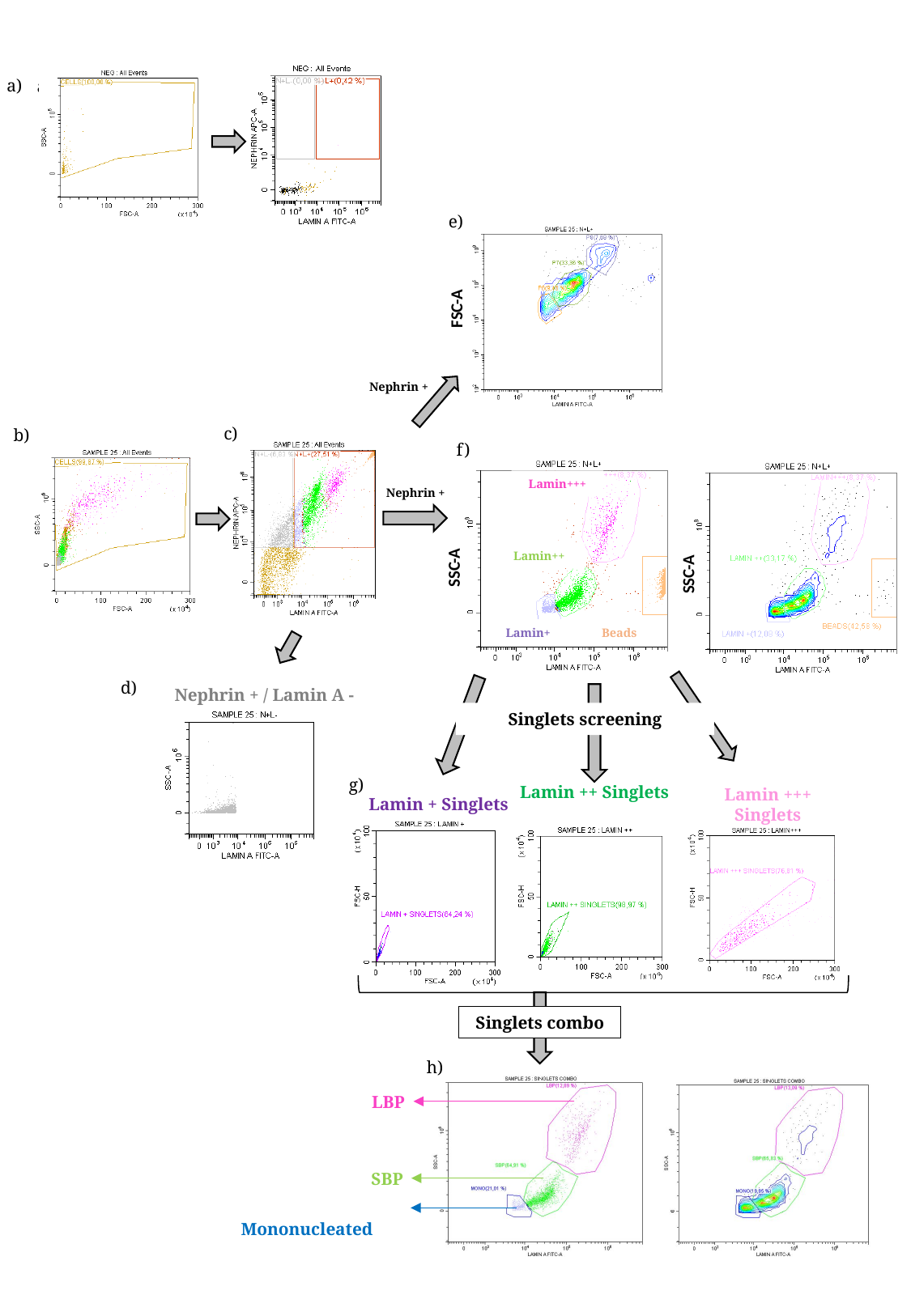

a)
a)
e)
FSC-A
Nephrin +
c)
b)
f)
Lamin+++
Nephrin +
Lamin++
SSC-A
SSC-A
Lamin+
Beads
d)
Nephrin + / Lamin A -
Singlets screening
g)
Lamin ++ Singlets
Lamin +++ Singlets
Lamin + Singlets
Singlets combo
h)
LBP
SBP
	Mononucleated

## Slide 3
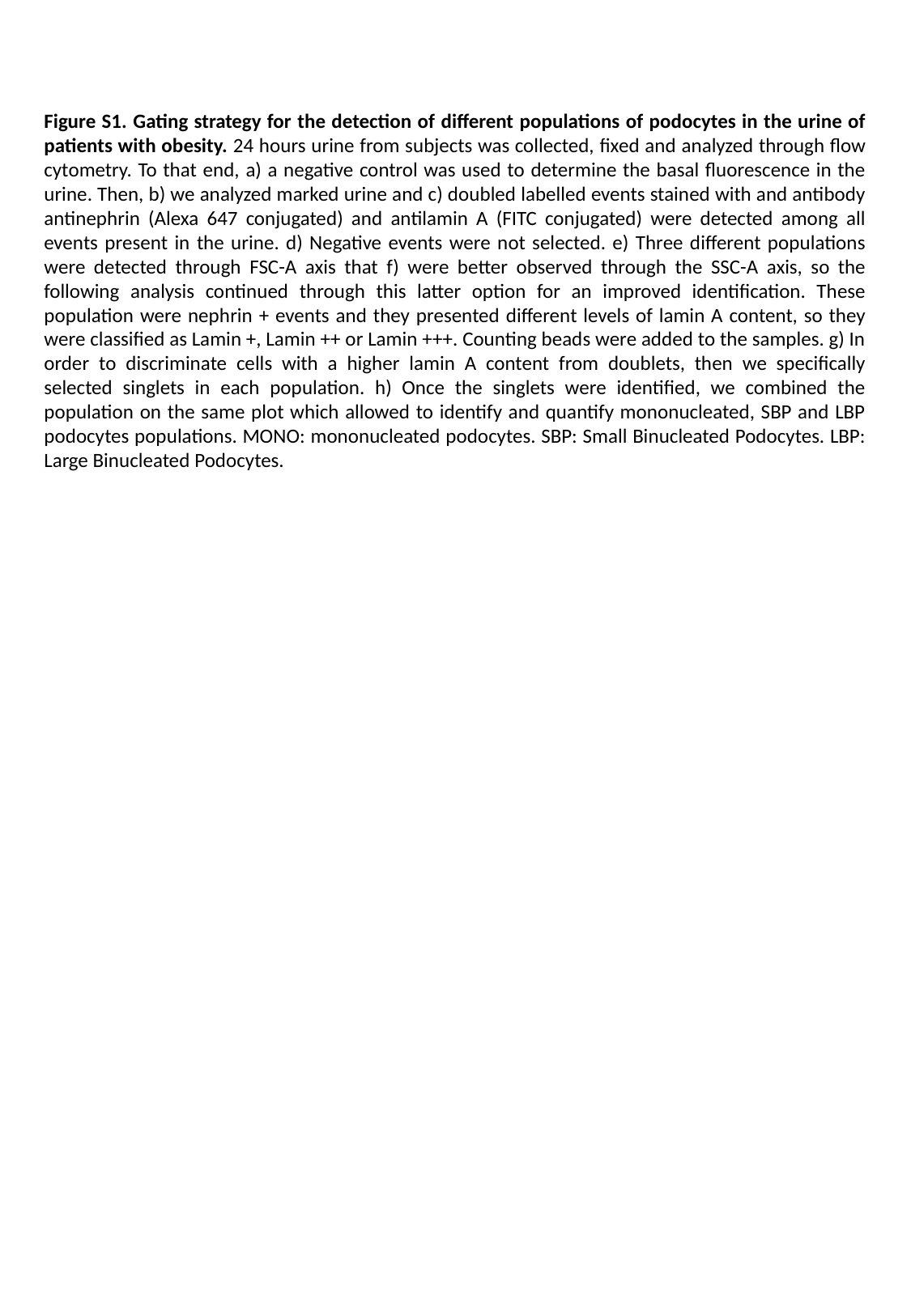

Figure S1. Gating strategy for the detection of different populations of podocytes in the urine of patients with obesity. 24 hours urine from subjects was collected, fixed and analyzed through flow cytometry. To that end, a) a negative control was used to determine the basal fluorescence in the urine. Then, b) we analyzed marked urine and c) doubled labelled events stained with and antibody antinephrin (Alexa 647 conjugated) and antilamin A (FITC conjugated) were detected among all events present in the urine. d) Negative events were not selected. e) Three different populations were detected through FSC-A axis that f) were better observed through the SSC-A axis, so the following analysis continued through this latter option for an improved identification. These population were nephrin + events and they presented different levels of lamin A content, so they were classified as Lamin +, Lamin ++ or Lamin +++. Counting beads were added to the samples. g) In order to discriminate cells with a higher lamin A content from doublets, then we specifically selected singlets in each population. h) Once the singlets were identified, we combined the population on the same plot which allowed to identify and quantify mononucleated, SBP and LBP podocytes populations. MONO: mononucleated podocytes. SBP: Small Binucleated Podocytes. LBP: Large Binucleated Podocytes.

## Slide 4
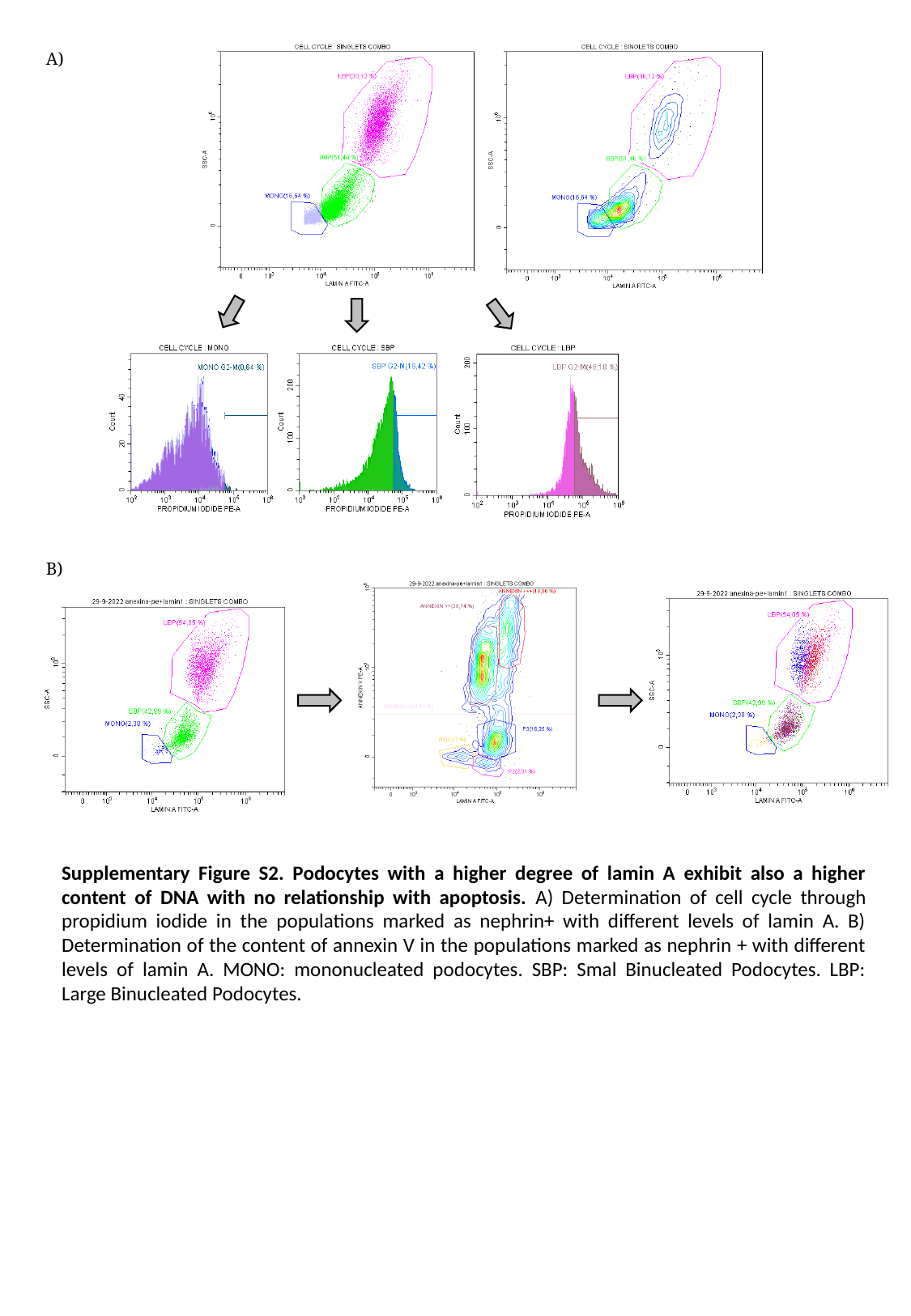

A)
B)
Supplementary Figure S2. Podocytes with a higher degree of lamin A exhibit also a higher content of DNA with no relationship with apoptosis. A) Determination of cell cycle through propidium iodide in the populations marked as nephrin+ with different levels of lamin A. B) Determination of the content of annexin V in the populations marked as nephrin + with different levels of lamin A. MONO: mononucleated podocytes. SBP: Smal Binucleated Podocytes. LBP: Large Binucleated Podocytes.

## Slide 5
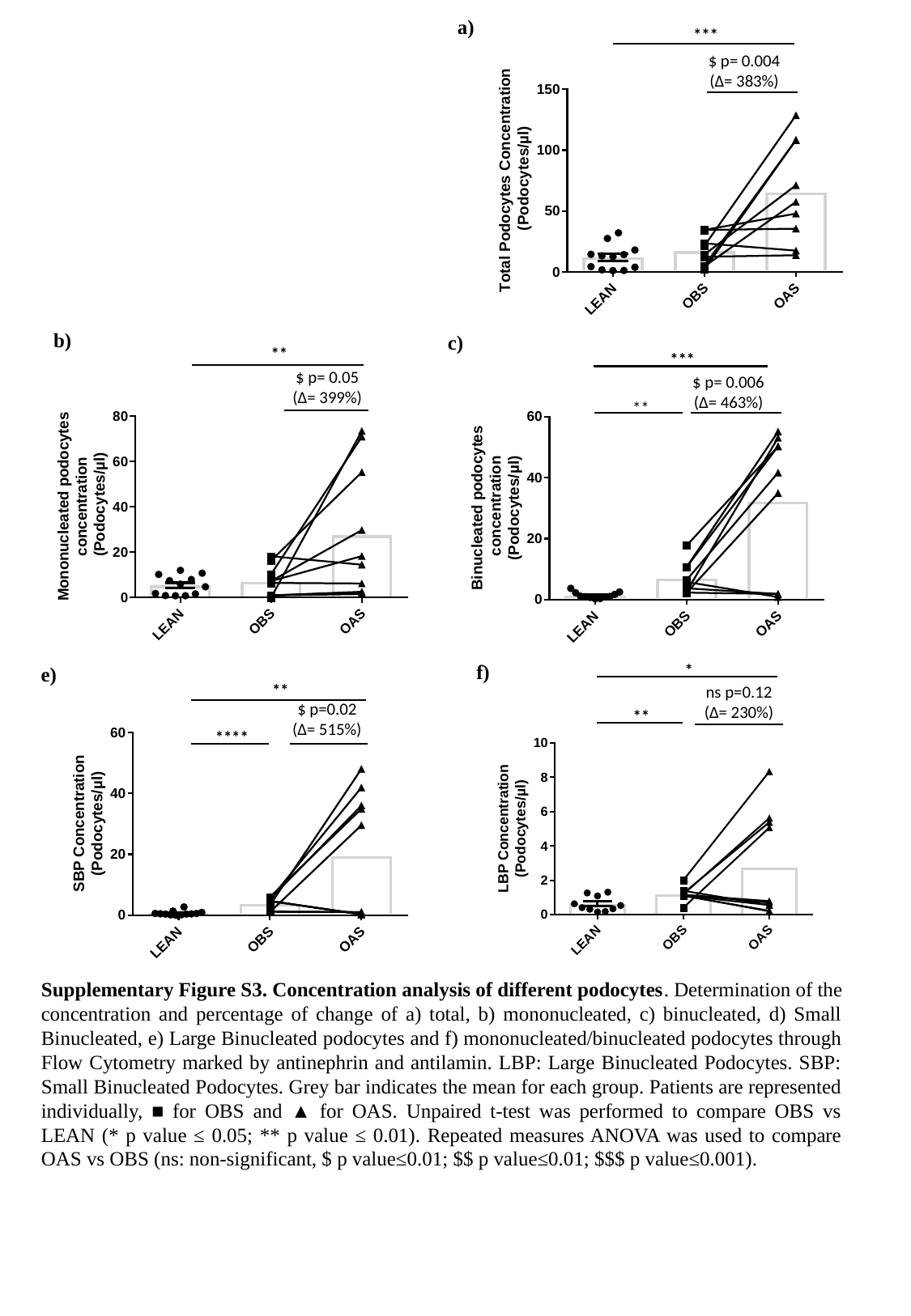

a)
***
$ p= 0.004
(Δ= 383%)
b)
c)
**
***
$ p= 0.05
(Δ= 399%)
$ p= 0.006
(Δ= 463%)
**
f)
*
e)
**
ns p=0.12
(Δ= 230%)
$ p=0.02
(Δ= 515%)
**
****
Supplementary Figure S3. Concentration analysis of different podocytes. Determination of the concentration and percentage of change of a) total, b) mononucleated, c) binucleated, d) Small Binucleated, e) Large Binucleated podocytes and f) mononucleated/binucleated podocytes through Flow Cytometry marked by antinephrin and antilamin. LBP: Large Binucleated Podocytes. SBP: Small Binucleated Podocytes. Grey bar indicates the mean for each group. Patients are represented individually, ■ for OBS and ▲ for OAS. Unpaired t-test was performed to compare OBS vs LEAN (* p value ≤ 0.05; ** p value ≤ 0.01). Repeated measures ANOVA was used to compare OAS vs OBS (ns: non-significant, $ p value≤0.01; $$ p value≤0.01; $$$ p value≤0.001).

## Slide 6
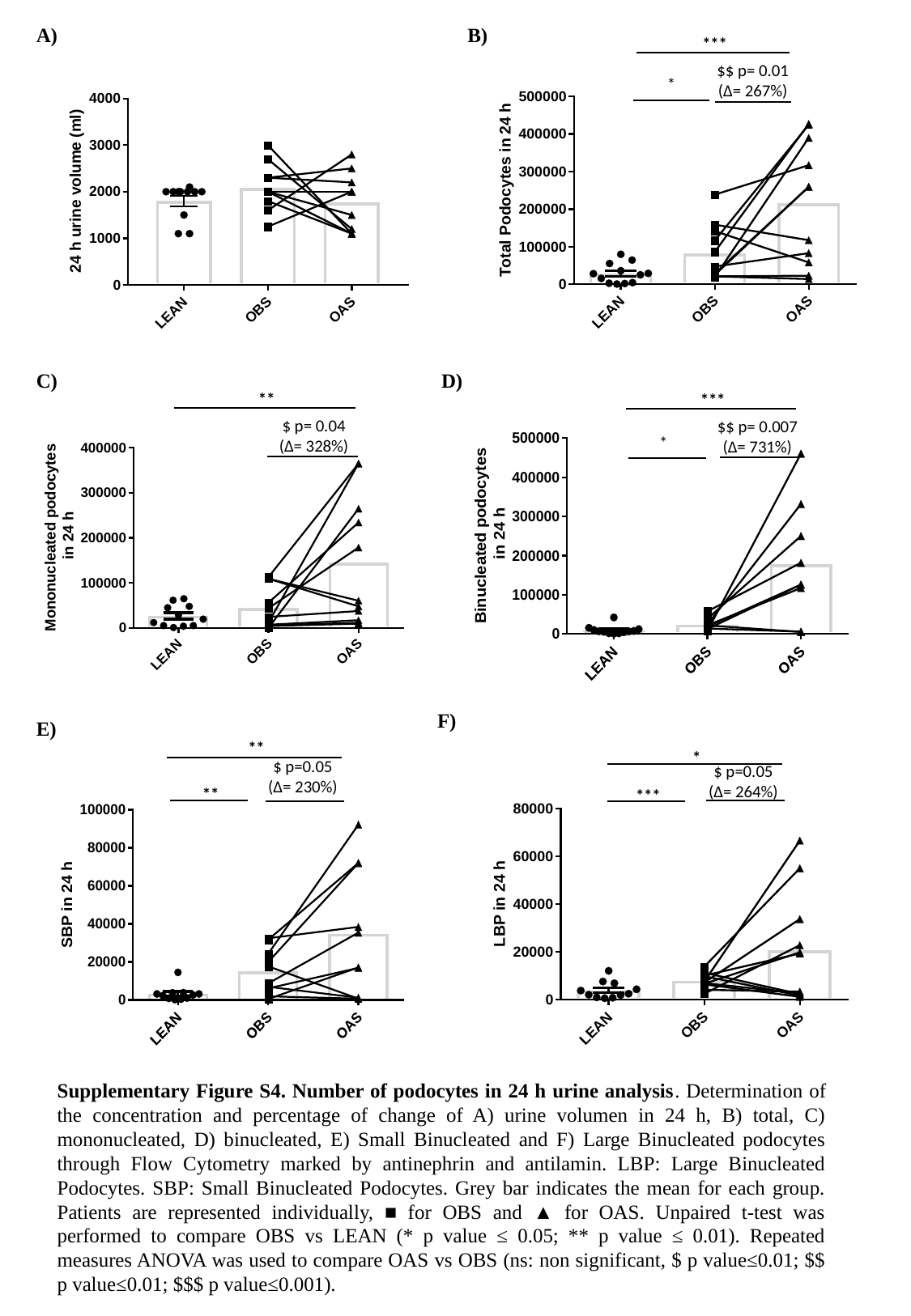

A)
B)
***
$$ p= 0.01
(Δ= 267%)
*
D)
C)
**
***
$ p= 0.04
(Δ= 328%)
$$ p= 0.007
(Δ= 731%)
*
F)
E)
**
*
$ p=0.05
(Δ= 230%)
$ p=0.05
(Δ= 264%)
**
***
Supplementary Figure S4. Number of podocytes in 24 h urine analysis. Determination of the concentration and percentage of change of A) urine volumen in 24 h, B) total, C) mononucleated, D) binucleated, E) Small Binucleated and F) Large Binucleated podocytes through Flow Cytometry marked by antinephrin and antilamin. LBP: Large Binucleated Podocytes. SBP: Small Binucleated Podocytes. Grey bar indicates the mean for each group. Patients are represented individually, ■ for OBS and ▲ for OAS. Unpaired t-test was performed to compare OBS vs LEAN (* p value ≤ 0.05; ** p value ≤ 0.01). Repeated measures ANOVA was used to compare OAS vs OBS (ns: non significant, $ p value≤0.01; $$ p value≤0.01; $$$ p value≤0.001).

## Slide 7
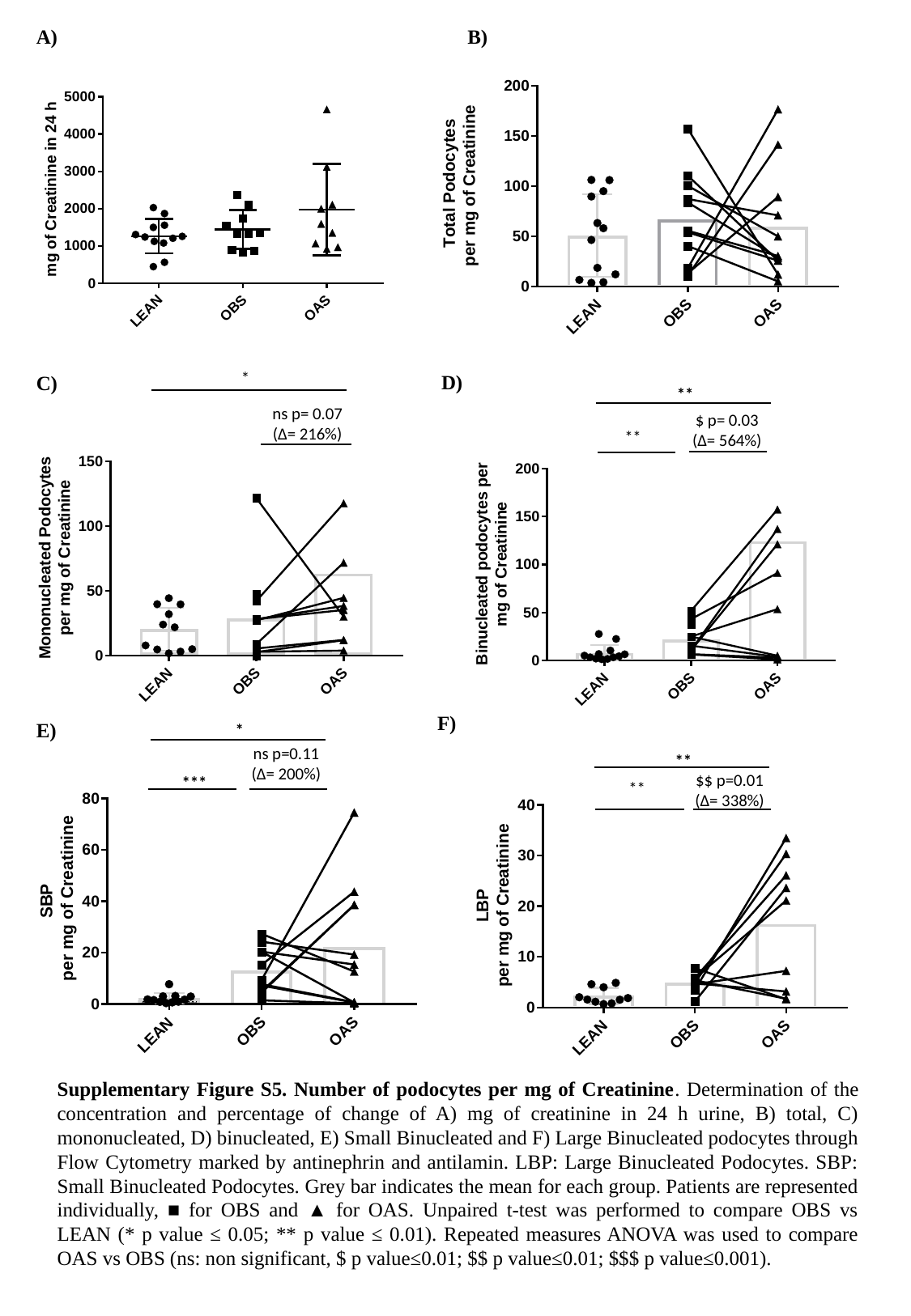

A)
B)
*
D)
C)
**
ns p= 0.07
(Δ= 216%)
$ p= 0.03
(Δ= 564%)
**
F)
E)
*
ns p=0.11
(Δ= 200%)
**
$$ p=0.01
(Δ= 338%)
***
**
Supplementary Figure S5. Number of podocytes per mg of Creatinine. Determination of the concentration and percentage of change of A) mg of creatinine in 24 h urine, B) total, C) mononucleated, D) binucleated, E) Small Binucleated and F) Large Binucleated podocytes through Flow Cytometry marked by antinephrin and antilamin. LBP: Large Binucleated Podocytes. SBP: Small Binucleated Podocytes. Grey bar indicates the mean for each group. Patients are represented individually, ■ for OBS and ▲ for OAS. Unpaired t-test was performed to compare OBS vs LEAN (* p value ≤ 0.05; ** p value ≤ 0.01). Repeated measures ANOVA was used to compare OAS vs OBS (ns: non significant, $ p value≤0.01; $$ p value≤0.01; $$$ p value≤0.001).

## Slide 8
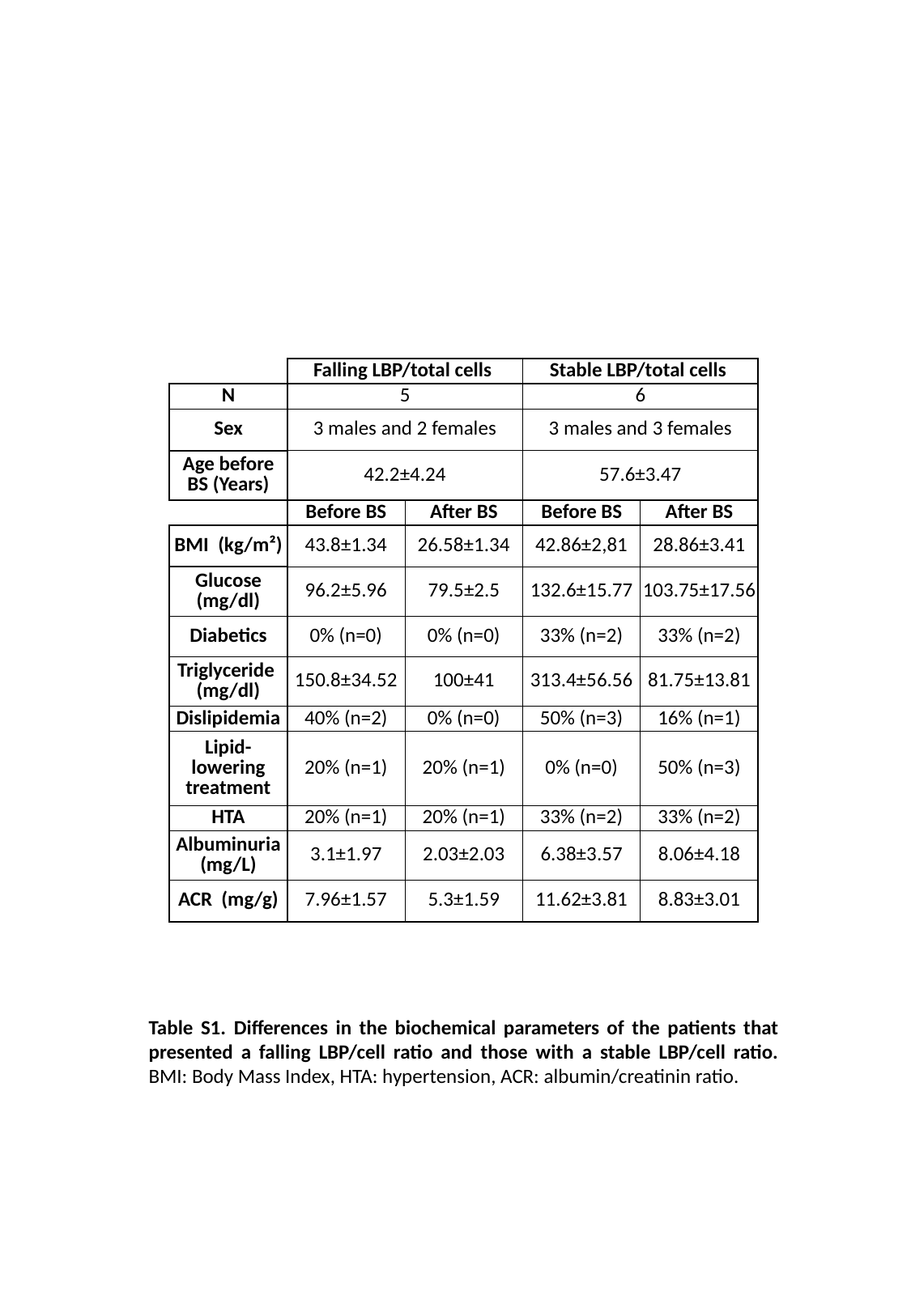

| | Falling LBP/total cells | | Stable LBP/total cells | |
| --- | --- | --- | --- | --- |
| N | 5 | | 6 | |
| Sex | 3 males and 2 females | | 3 males and 3 females | |
| Age before BS (Years) | 42.2±4.24 | | 57.6±3.47 | |
| | Before BS | After BS | Before BS | After BS |
| BMI (kg/m²) | 43.8±1.34 | 26.58±1.34 | 42.86±2,81 | 28.86±3.41 |
| Glucose (mg/dl) | 96.2±5.96 | 79.5±2.5 | 132.6±15.77 | 103.75±17.56 |
| Diabetics | 0% (n=0) | 0% (n=0) | 33% (n=2) | 33% (n=2) |
| Triglyceride (mg/dl) | 150.8±34.52 | 100±41 | 313.4±56.56 | 81.75±13.81 |
| Dislipidemia | 40% (n=2) | 0% (n=0) | 50% (n=3) | 16% (n=1) |
| Lipid-lowering treatment | 20% (n=1) | 20% (n=1) | 0% (n=0) | 50% (n=3) |
| HTA | 20% (n=1) | 20% (n=1) | 33% (n=2) | 33% (n=2) |
| Albuminuria (mg/L) | 3.1±1.97 | 2.03±2.03 | 6.38±3.57 | 8.06±4.18 |
| ACR (mg/g) | 7.96±1.57 | 5.3±1.59 | 11.62±3.81 | 8.83±3.01 |
Table S1. Differences in the biochemical parameters of the patients that presented a falling LBP/cell ratio and those with a stable LBP/cell ratio. BMI: Body Mass Index, HTA: hypertension, ACR: albumin/creatinin ratio.
